# Extra Supplement—The Nursing Mirror

**Published:** 1890-11-29

**Authors:** 


Being the Extra Nursing Supplement of "The Hospital" Newspaper.

All Contributions for this Supplement should be addressed to the Editor, The Hospital, 140, Strand, London, W.C., and should have the word "Nursing" plainly written in left-hand top corner of the envelope.

### En Passant.

Religion at Leicester.—Leicester Infirmary has narrowly escaped from another battle on the question of nurses and their religious duties. A branch of the Guild of St. Barnabas was inaugurated, with Miss Rogers as local superior, and a special service was held in the infirmary chapel. Fifteen subscribers to the infirmary wrote a protest to the committee, and the chaplain undertook that the service should not occur again, and expressed his regret that he had permitted the nurses to hold it. Of course in a public institution it is wise that the religious services should be as free as possible from party teaching, and Leicester has already suffered severely from permitting its nurses to bind themselves, save by the rules of the institution.

Short Items.—Mrs. Martin, Nurses' Home, Grange Mount, is secretary of a committee which has been founded to erect a suitable memorial to the late Nurse McCoy, who died on duty at the Birkenhead Fever Hospital.—Nurse Gough, who took the last Examination Question prize, wishes us to state that she is connected with the Nurses' Home, Upper Parliament Street, Liverpool.—Mrs. Alice Hayes visits the Calcutta Leper Asylum every week, and reads to the patients.—Rushington, in Sussex, is to have a village nurse.—Mrs. Van Buren has resigned the matronship of the Leavesden Asylum.—Dr. Boddie is giving a course of nursing lectures in Edinburgh.—Two lady nurses have gone to Africa with Dr. Smythies.—We have received the names of some homes and hospitals which persistently work their nurses for fourteen hours; also of one hospital which never gives its nurses either puddings or green vegetables.

A Cornish Story.—In the parish of Camborne, the centre of the tin mining industry of Cornwall, live over 8,000 miners, whose average rate of wages is three pounds per month. Little as this may appear to a stranger, it is possible for a man to bring up his children in decency, and some little comfort on these wages; for in this far away district rents are low, living cheap, and the extreme mildness of the climate causes the winter to press much less hardly on the poor than in most other parts of England. But unfortunately, the conditions under which tin mining is necessarily carried on are prejudicial to the health of the miner, and induce, at an early age, many forms of consumption or "miner's sickness," as it is called by those who suffer so fearfully from this fell disease. Accidents, too, some from machinery, some from the running in of ground or falling of rocks, or premature explosions in blasting, all tend to make the miner's life a precarious one, and the three pounds per month on which it is possible for him to bring up his family, leaves small margin for saving against the proverbial rainy day. To meet this difficulty, about eighteen months ago, a few ladies determined te make an effort to supply the town of Camborne with a trained nurse, whose services should be devoted to those unable to pay for, or obtain such services for themselves. A committee of ladies was formed, whose appeal to the public was so generously and promptly responded to, that in April, 1889, Nurse Lloyd began her duties as district nurse; during the year she attended 125 patients regularly, and to these she paid 2,263 visits. At a public meeting held in April, 1890, it was decided to engage a second nurse and extend the work to the villages lying around Camborne. The nurses are provided with hospital requisites for lending, and where necessary, supplying medical comforts, such as beef tea, milk, and wine. This charity is supported with equal generosity by the Church of England, Dissenters, and Roman Catholics.

Another Hostel.—Miss Wood's notion of a hostel for nurses is being widely taken up; the latest addition to these useful institutions is "Oakfield," Forest Hill, S.E., which has been started by Mrs. Robinson, at one time superintendent of the Kent Nursing Institution. We think Mrs. Robinson makes a mistake in combining a home for nurses and a private hospital; the nurses can never enjoy themselves so freely between cases when they are in the same house with invalids, and there is always the chance of extra work being thrown on them.

Nurses' Missionary Association.—"Them as invented nurses and wadding deserve to have a crown of glory." This is the opinion of a woman in one of our great coal-begrimed cities; and the sentiment will be echoed by thousands who value truth more than the way it is expressed. "God bless them nurses," comes from the heart of the people. Whether in hospitals or infirmaries, convalescent homes, or in districts, nurses have the power and opportunity of influencing others, which is much to be envied. If medicine has been described truly as "the handmaid to the preaching of the Gospel," how shall we describe nurses? During the last few years we hear that the value of doctors and nurses in connection with all missions has met with increasing recognition. If this is so in England, how much more must it be in foreign parts where deeds of mercy and love are the only means of getting at those whose language and life are so apart from ours? We hear daily of nurses being wanted for foreign missions. The Nurses' Missionary Association is established to send nurses abroad by the help of nurses at home. By paying one shilling a year any nurse or nurse's friend can become a member of the association. This is the only subscription required. As a member she will receive news of what is being accomplished, and if she finds herself able to help in any other way, work will be suggested, though nothing beyond a shilling a-year and her prayers will be requested unless she chooses. The ways of working will be as follows: (1) She may become a helper—that is, she may ask others to join and receive their payments, which she would forward to the secretary, with their names, once every three months; (2) she may collect from her friends and acquaintances funds for the association; (3) she may send needlework of any kind to be sold, the secretary arranging for the sale of it; (4) she may supply lint, bandages, "wadding," or old linen to be sent abroad; (5) she may be put in communication with a nurse working abroad and supply her with papers, periodicals, or any articles of comfort or pleasure which she may require. The funds of the association go towards furnishing the salaries of nurses in foreign missions, or supplying nurses with such necessaries as they require. The association has only very lately been started. It has already 700 members. They have been able to supply beds and bedding to the new hospital at Mahanora; to supply the salary of a native nurse at Peking, and to give £20 a-year towards the nursing fund of the Central African Mission. Nurses are wanted in Cyprus, in Japan, at Ameritza, and other stations in India, as well as Burmah, in Peking, and, in fact, nearly everywhere there are mission stations, but from the places named there are definite calls for help. Not only are we asked to supply funds for the nurses' salaries, &c., but to stir up missionary zeal in nurses that they may come forward and give themselves to missionary work. The secretary of the Nurses' Missionary Association is Miss D. M. Image, 53, Gloucester Street, S.W., who will be glad to receive the names and subscriptions of new members as well as donations for the association.

### Lectures on Surgical Ward Work and Nursing.

By Alexander Miles, M.B. (Edin.), C.M., F.R.C.S.E.

Lecture V.—WOOL AND LINT.

Varieties of Wool.—Wool is very largely used in surgery for padding splints and other appliances, as well as a part of almost every dressing. There are different varieties of wool—some plain, others charged with antiseptic agents.

(1) Plain Wool.—This is ordinary cotton-wadding made up in sheets about half an inch thick, and having one side covered with some kind of paste which keeps the sheets together. This wool is not charged with any antiseptic, nor is it absorbent, and must therefore never on any account be used in dressing an open wound. Nor is it allowable to use it for padding splints in the setting of a compound fracture, or for any part connected with a discharging wound. For these purposes antiseptic wool of one kind or another must be employed.

Plain wool may only be used for padding splints for a simple fracture, or for any purpose where you are dealing with unbroken skin.

Always peel off the paste backing before using the wool, as it interferes with the escape of the skin secretions, which are apt to cause irritation when retained.

(2) Corrosive Sublimate Wool.—This is fine white cotton wool, which has been rendered antiseptic by being impregnated with corrosive sublimate. It is used in the dressing of all open wounds, and for padding splints and other appliances which are to come near sources of discharge. The advantage of corrosive sublimate wool, of course, lies in its being antiseptic, but it must be borne in mind that the pus and blood render corrosive no longer antiseptic by forming an albuminate of mercury. Hence, so soon as ever a dressing gets soaked with discharge it is no longer *anti*septic, but only *a*septic, and should organisms gain access to it, it will soon become *septic*. The practical application of this is, that when using corrosive wool you should keep a sharp look out on the discharge, and never allow it to get through the bandage. If it do get through in spite of your watchfulness, *at once* re-dress the wound. If you delay, the chances are the wound will become septic, as pus in a warm, moist pad of wool forms an excellent nidus for germs, and germs never lose an opportunity of establishing themselves.

(3) Wood Wool.—This is made by subjecting chips of pine wood to certain chemical processes, by which the oils and resins are removed, and the remaining substance is rendered very absorbent. It is charged with corrosive sublimate to make it antiseptic, and has the advantage of being much more absorbent than ordinary corrosive wool. When saturated with discharge it, too, loses its antiseptic properties.

(4) Salicylic Wool is fine cotton wool charged with salicylic acid. Two strengths of the antiseptic are used, the stronger containing 10 per cent. by weight, the weaker 3 per cent. The excess of powdered acid which comes off the wool is apt to irritate the eyes and nasal mucous membrane of those around, setting up in some people a violent choriza. For this reason it has been given up by many.

It will thus be seen that each of the forms of wool I have mentioned has certain disadvantages: some are irritating, others are rendered non-antiseptic by discharges, and all are expensive. We still want a cheap, efficient, non-irritating, and absorbent substance to take the place of wool. Several of these are at present on their trial, but, meanwhiie, perhaps the best substance available is corrosive wool.

There are one or two points of importance in preparing the wool for your dressing-tray. Wool must never be allowed to lie exposed to the air, and its inevitable dust and germs. It is best kept clean by being placed in well-closed tin boxes, somewhat larger than large biscuit boxes; or failing these, it should be kept rolled up in the thick paper in which it is sold. As it comes from the chemist it is in long thick rolls, each weighing about a pound. You will find it convenient to cut these rolls into pads about a foot square, and place them one above another in your boxes. As wool will only tear in its *long axis*, it will be easier, and will waste less wool, if you cut the rolls *across* with scissors, rather than attempt to tear it.

Lint.—(1) Plain or Surgical Lint is a soft material, made by scraping old linen cloth, and is used as a dressing in certain wounds, &c. It is non-antiseptic, therefore its use is much restricted. It is often covered with antiseptic ointments, oils, &c., and applied to open sores. The two sides of lint are different—one is plain the other is woolly—and the question is often asked, which side should go next the wound? It is very much a matter of taste. There are certain points, however, to attend to. You will find that you can spread ointment more evenly and with greater ease on the plain side. Therefore do so. The plain side is less apt to stick into a sore, and the dressing more easily removed when you put it down. On the other hand, when applying lint to an unbroken surface, the "fluffy" side is the softer, and more absorbent of skin secretions.

(2) Boracic Lint is ordinary surgical lint which has been soaked in a hot, saturated solution of boracic acid, and then hung up to dry. It is thus rendered an efficient antiseptic, and the boracic acid being non-volatile it retains that property. It should, however, never be applied next a wound *dry*, as it has been found that its germicidal power is greatly increased by moisture. It is usually tinted pink by means of litmus, to distinguish it from ordinary lint. It is also covered with the excess of boracic acid crystals deposited as the lint cools. It is exceedingly absorbent of discharge. All round it is a most valuable material, being cheap and handy, only requiring to be moistened to furnish a fairly efficient antiseptic dressing for all minor injuries, such as cut hands, scalp wounds, small ulcers, and so on. It also forms a good deep dressing in more important cases. Another use to which it is often put is to cover the limb before applying a plaster case or extension. Boracid lint is preferred to plain for this purpose, as the former is toxic to fleas and other body insects—a point of great importance in our out-patient departments.

Advantages.—1, Cheapness; 2, efficient antiseptic; 3, almost universal applicability; 4, very absorbent; 5, toxic to fleas, &c.

Disadvantages.—None.

*(To be continued.)*

### The Glasgow Sick Poor and Private Nursing Institution.

Her Royal Highness Princess Louise, accompanied by Sir Archibald Campbell, of Blythswood, on November 19th paid a visit to the home of this association, of which she is patroness. H.R.H., after conversation with the Lady Superintendent (Miss Wood) and District Superintendent, Miss Whyte (both trained at Westminster), and the district nurses, expressed herself as very much pleased indeed with the nurses and the records of their work, as well as the general management and system upon which the home is conducted.

### Death in Our Ranks.

Miss Neville, one of Lady Rosebery's nurses, died on Monday of typhoid fever in the Edinburgh City Hospital. She belonged to the Victoria Nursing Association.

### The Trained Nurse in the Nursery.

My Dear Friends,—I am asked by your friend and mine, the "Editor," to tell you from my experience what is essential in a trained nurse, besides her hospital training, to make her a successful nurse in a private nursery.

I am rather afraid of summing up; it seems as though a nurse, entrusted with the sole care and training of little minds, apart from the body (when we realize that the children of to-day hold the future welfare of nations, it may be, in their hands), needs to be perfect. But, alas! that being impossible, I must confine myself to what is absolutely needful.

A nurse must be *strictly truthful*. I shall never forget a little child of four, who had heard he was going a journey next day, and, as children will do, got very excited over the prospect. At last, getting a little impatient of the noise, I said, "How do you know you are going? Little boys who make a noise like that don't always get what they want." I meant to put the child off, but not to tell him an untruth. Next morning the child said to me in a very reproachful voice, "Nannie, you told me a story yesterday; you said I was not going." Now I knew I had not *said* it, but I was conscious of wishing to imply it. That child's voice and look has been with me daily since. One is so apt to say things to children without thinking at the moment that the young child is capable of seeing our motives and testing our truth beyond what we think.

A nurse must be *honest*. She is very much alone, can do in a great measure what she likes in portioning out her time, &c.; and we ought to remember our time belongs to our employer, unless we arrange for a special portion for ourselves. It must be bad for children to see work put under the table, a book thrust out of sight, when the mistress makes an unexpected appearance in the nursery. Children will notice these things, and it is not honest to pretend we are doing nursery work when otherwise employed. I mention this as having seen it take place in a nursery of good social-standing.

A nurse must be *clean*. I hope my friends will not take offence, but I have seen two and three children bathed in the same water, diapers put under a bed unwashed, and by a so-called trained nurse; bottles left for another time, dirty, &c. These things will never answer in a private nursery. The under-nurse will say, "Nurse does this, why should not I?"

A nurse must be *respectful*. I myself sent a nurse to see a lady this week about the situation of nurse, and she did not get the place because she seated herself in the drawing-room without giving the lady time to ask her; she did not say "ma'am," or, in fact, wait to inquire what the lady wished, but stated, in a very abrupt manner, what *she* wanted. Civility begets civility, and when we remember that even a duchess has to say "ma'am" to the Queen, and that we should never think of speaking to our matron otherwise than as matron or ma'am, to our doctor as sir, why should we mind doing it in private work?

A nurse must have nice manners, must be gentle and motherly to the little children; and, remember, in the nursery it is most important to have the soft answer ready at all times. A nurse must be *discreet*. She will naturally, living so much in the midst of the family, hear and see many things not intended for strangers' ears, therefore she should make a point of never repeating the simplest thing.

A nurse ought to know how housework is done, that she may be able to teach her maid thoroughly, should it be necessary. Most ladies require a nurse who can sew nicely, or at least be able to keep the children's things in repair. If a nurse aspires to a grand nursery, she should know the proper treatment and feeding of a wet nurse; and, of course, every nurse must understand the washing, dressing, feeding, and general management of an infant from the month, as well as the disorders of teething, weaning, &c. As a trained nurse, she will be naturally the referee in all sickness in the household. She will be appealed to in any accident or emergency. In many ways she will be the prop of the house generally, and, as a woman, her mistress's personal friend and general adviser with her family; and, if she has tact enough to wait and work, will, in time, find herself the most important person in the house; but, like her babies, she must crawl before she can walk, and walk before she can run.

My dear colleagues, I am afraid many of you will say, what a sermon! Of course we are all obliged to be truthful, honest, sober, gentle, and discreet; our hospital school demands that before we get our training. Yes, dear sisters, I am fully aware of that, being a hospital-trained nurse myself of many years' standing. Let me beg of you to remember, before taking up this, a new branch of work in our profession, that the influence and character of his nurse, under God, have given the turn to the many great and good men who, from time to time, have done so much for our country, and let us never say, It does not matter, he is only a baby!

Next week I will describe a day's work in an ordinary nursery, and let me tell you it is a happy life. There is much to interest, and much to do. A nurse can remain in a family for years; in fact, never grow too old. Her nurslings will love her. But—yes, there is a but, and a big but—at first the life is trying, after the excitement of a hospital. Your nursery is liable to invasion from master, mistress, and their friends at inconvenient times, and you have been accustomed to a room strictly your own; you get no time off duty, you find the life very humdrum, you don't like being seen wheeling the baby's perambulator, and the under-nurse must be left at home sometimes on extra cleaning days, you don't like wearing print dresses all day; in fact, you are afraid of being called a servant. Yet are we not all servants who serve in any capacity? But let us remember in our employer's house we may be called a servant, though my experience would lead me to say a good nurse will be considered more friend than servant. We must all remember that nothing but dishonour can ever make us any less our father's daughter in whatever position we may have been born.

I can hear some of you ask what the social position of a trained nurse in an ordinary household would be. I answer, quite equal to that of an ordinary governess; and, in spite of drawbacks, there is much pleasure to be got out of the life. Most families now-a-days go to the sea or Scotland for some weeks in the year. A nurse gets nice drives to vary the daily outings, she lives in pleasant rooms, and as everything must be of the best for the bairnies, it follows it must be healthy for the nurse also.

My sisters, I write from experience. I am one of you; in many ways in the nursing world I have been prosperous. I love hospital and institution life, yet, not being blessed with good health, I have had periods of rest, during which I have often taken temporary work in nurseries, and I like it. Ladies frequently say to me, "I would give anything for a good trained nurse in my nursery; but they won't do the work." So I want you all to face the fact that it is work, but I am sure not too heavy work for any woman in fairly good health to undertake between the age of forty and fifty-five.

If any of you are willing to undertake the duties, an advertisement in The Hospital, *Christian*, or *Morning Post*, stating your qualifications, will, I am sure, bring you its certain measure of answers, from which you must each choose for yourself the one most suitable. To you who have the courage to begin this new work, I give my heartiest sympathy and thoughts, and remain, yours faithfully,

Sister Grace.

### Nurses on Their Travels.

NOTES FROM AUSTRALIA.

(*By Our Own Correspondent.*)

The Commission on Charities has turned its attention again to nursing, and has examined the matrons of most of the large hospitals of Victoria.

Mrs. Strong, matron of the Alfred Hospital, gave information as to the system of nursing carried on at that institution. No difficulty, she said, was experienced in getting sufficient pupils for training. The nurses had lately been relieved of menial work, and the practice had been stopped of sending out any but certificated nurses.

Miss Eliza Morley, matron of the Austin Hospital, said that her experience of trained nurses led her to believe that they did not care about attending chronic cases. In the Austin Hospital, therefore, she believed untrained nurses were preferable to trained ones. The nurses worked 14 hours a day, and got £35 a year as wages.

Mrs. Muffatt, matron of the Homœpathic Hospital, stated that the nurses at that institution were well provided for, having a sitting-room and dining-room and the use of a piano and library.

Miss Alice Martelli, trained nurse, said that nine hours was quite long enough for a nurse to work. She had known of nurses working as many as sixteen hours a-day, with half an-hour for meals. It would be advisable to have a general board for the examination of nurses, and to give diplomas. If a woman worked more than nine hours in the day, the patients under her charge must suffer. She disagreed with the idea that untrained nurses were suitable for incurables. In the first place an untrained nurse would not know how to lift a patient. If the remuneration were increased at the Austin Hospital, the services of trained nurses might be obtained. Nurses had a good deal of responsibility, and without a good nurse a doctor could do very little good. Considering the nature of their duties, head nurses should be paid £80, and assistant nurses £50 a-year. There should be a central home for nurses for the convenience of the general public.

The evidence of Miss Morley is a sign of how backward nursing is in Melbourne. Miss Morley is herself untrained to all intents and purposes, and hence the explanation of her extraordinary statements.

In our last "Notes from Australia," mention was made of the intention of two ladies to provide a private hospital for typhoid patients; the accommodation at present in the hospitals in Melbourne is not nearly sufficient. Mrs. Strong, who has been so successful a matron of the Alfred Hospital for many years, and Miss Martelli, formerly head nurse at the Melbourne Sick Children's Hospital, more recently matron of the hospital at Maryborough, are undertaking this arduous work. They have both had great experience, and are extremely well fitted for the charge of typhoid patients. The house is chosen well in a healthy suburb, and very conveniently situated for both trams and railway. It is called "Rokeby," and is in Redan Street, Windsor, Melbourne. They hope to be ready for the "season of typhoid," which, alas, is already beginning, and from which we are never entirely free. What a comfort to think that if one must have typhoid, one could be placed in such capable hands. No doubt many whose houses are so unsuited for nursing in, as the generality of Australian houses are, will avail themselves of this opportunity when need arrives.

Here is a note from the last meeting of the Alfred Hospital Committee: "The matron, Mrs. H. T. Strong, forwarded her resignation of the position held by her for ten years. Her reason for doing so was that she was, in conjunction with Miss A. Martelli, matron of the Maryborough Hospital, about to establish a private hospital. The resignation was accepted with regret. The building committee recommended certain improvements to the nurses' dining-room." It is to be hoped a worthy and trained successor will be found to Mrs. Strong.

### The Institute of Medical Electricity.

In his lecture on the 8th inst. Mr. Lawrence spoke of the importance of measuring the amount of electricity applied to a patient. It was of little good to judge by the number of cells in use, for the cells of one battery differed greatly from another in strength and were also affected by atmospheric and other circumstances. Before application the current should be tested by a galvanometer; these machines were usually constructed with a magnetic needle, freely suspended or pivoted in a coil of wire. The current passing through the coil caused the needle to deflect strongly, the deflections being shown by a dial with numbered divisions. Another way of measuring electricity was by the decomposition of water in a glass tube, but this method was little used. As regarded the alternative current there was, as yet, no machine for correctly testing the electro-motive force. The smallest galvanometer was marked in milliamperes, and even a milliampere of electricity could not be borne by a patient when applied by means of a coil. The best way to judge was to notice the amount required to produce muscular contraction by means of the centimetres marked on the machine. On the following Friday Dr. Harries discoursed on the nervous system, commencing with the brain. The motor nerves, proceeding from the front part of the spinal cord, controlled the movements of the muscles, while the sensory nerves coming from the back part of the cord were the nerves of touch or feeling, and conveyed sensations to the brain. The centres of the sympathetic system consisted of a chain of knots or ganglia of nerve matter lying on either side of the vertebral column; from these ganglia nerves proceeded to all the internal viscera, and, to a great extent, controlled the functions of digestion, nutrition, circulation, and respiration.

### Everybody's Opinion.

[*Correspondence on all subjects is invited, but we cannot in any way be responsible for the opinions expressed by our correspondents. No communications can be entertained if the name and address of the correspondent is not given, or unless one side of the paper only be written on.*]

TETANUS.

"Nurse M. F." writes: In reply to the question of "J. G." (No. 9), perhaps the following particulars may be of interest. In June, 1883, a married woman, aged thirty-five, was brought into C—— infirmary in a state of tetanus. She had on the outside of the elbow a recently-healed wound which had been caused a fortnight before admission by a broken jug being thrown at her in a quarrel. The patient when admitted was perfectly rigid, and the jaws were firmly locked. About an hour after being put into bed she had a spasm, during which the body was arched backwards, so that only the head and heels touched the mattress. She had lost several front teeth, so that a spoon could be put into her mouth, and I found that she could swallow, though with difficulty. The spasms continued to be both frequent and severe. She did not sleep, but took nourishment well. The doctor told me to give what spoon-food I thought best. I made some oatmeal porridge with milk only, rather stiff, and found she swallowed it more easily than fluids. She took this food at first in very small quantities, but at the end of the first week she was taking about three good breakfast-cupfuls, in the twenty-four hours. After the first three days she was moved into a private ward, which was kept warm, shaded from the light, and perfectly quiet. Spasms became less frequent, but she was very stiff, and could not turn in bed or raise herself from the pillow. The doctors all thought very badly of her chances of recovery, and the priest gave her the last sacraments. However, from about the tenth day after her admission there was a very gradual but steady improvement in her condition. The spasms disappeared first, and then the stiffness of the jaws and body gradually relaxed, and at the end of a month she was able to be dressed, and was taken home in a cab. She had no further bad symptoms, and was soon perfectly recovered. The medical treatment of the case was the same all the time she was in hospital, namely, a morphia injection every six hours, an ordinary chloral draught every night, and a tonic dose taken every four hours. It was iron and quinine, I believe. The bowels were moved at regular intervals after a dose of castor oil. Several other cases of lock jaw occurred in C—— infirmary during the spring and summer of 1883. An ovariotomy case had tetanus some weeks after the operation, and recovered in about five weeks' time. I remember the fact of her recovery, though I did not nurse her myself.

### LADIES ON COMMITTEES.

"A. E. S." writes: It appears to me that matrons and sisters-in-charge have a very good reason for objecting to "ladies on committees." A matron who cares for her nurses will only too willingly "furnish nurses' rooms, provide comforts for them, and decorate wards" if she can get "her want-book" signed by the chairman. It is simply a matter of money, and when money is so hard to get for every-day requirements, it does not need telling that luxuries are utterly "non-getable." Why cannot outside ladies give nurses comforts, provide decorations, &c., at once, without waiting until they are "elected to sit on committee"? Gifts are thankfully received, though, after many years of hospital work, I can truly say the said gifts consist chiefly in old rubbish, and that a very small percentage of what is worth anything finds its way to the hospital. I never had any present given for either nurses' bed or sitting rooms, and as to "contributions from 'crusty old bachelors,'" why in the world cannot a lady beg without being a committee woman? I still stick to my former expressed opinion, that we can sweep our own door-step; and if we cannot, well, replace us with someone who can; and that no servants will stand more than one "overlooker."

### SHALL WOMEN PROPOSE?

"F. M. H." writes: Excuse me, but anent "The Doctor's" article "Can Science Resolve the Doubt?" surely it is the female nightingale that sings, is it not? What says the poet:

> "And *she* that doth most sweetly sing;
> Sings in the night when all things rest."

She evidently charms, not chooses her mate! Let all women, I say (whether nurses or not), follow the example of the nightingale! [Poets are not famed for accuracy; we regret to say that the doctor was correct: it is the male bird that sings.—ED.]

### MALE NURSES.

THE SECRETARY OF THE HAMILTON ASSOCIATION writes: I must demur to the statement of "J. E. C." that it is for mental and epileptic cases only that male nurses are in much demand. We continually send out male nurses to other kinds of cases. If "J. E. C." has any practical experience of nursing, he must be aware that it is an occupation in which the amount of employment fluctuates considerably. It is, therefore, not surprising that on one particular date fifteen of our men should have been unemployed; I may add that some of these were off duty by their own wish.

### Appointment.

MISS RUDD, of Little Musgrave, Westmoreland, has been elected nurse for the parish of St. Andrew's, Penrith. Miss Rudd was trained at the London Hospital, and has since had several years' experience in both medical and surgical nursing. Her last appointment was in Perth, as district nurse, where she worked with the greatest acceptance and success.

### Notes and Queries.

### Queries.

(15) *Nightdresses.*—I should be glad if some one would tell me what kind of nightdresses the children at the Great Ormond Street Hospital wear, as I wish to make a few to send at Christmas. I think it is some kind of flannelette, but I don't know if it is coloured, or about what price it should be.—*A Lover of Children.*

(16) *Cancer and Electricity.*—Having read in some paper a short time back that cancer could be cured by means of electricity, I should much like to know if this is really the case, and where, or in what hospital it has been tried?—"*Senex.*"

(17) *Notice.*—What is the shortest notice to leave, necessary to give at a permanent case?—*Lincoln.*

### Answers.

(8) *Diet.*—Try Rizine; also Edward's Desiccated Soup (white). It is easy of digestion, and generally liked.—*Doris.*

(10) *Insurance.*—The hospital is not liable for a nurse's private property, such as bonds, which may be destroyed by fire. The nurse must insure them herself if she thinks it necessary.

(15) *Nightdresses.*—Children's Hospital nightdresses ought to be made of calico, not flannelette. It is usual for the small patients in our London hospitals to wear a white flannel vest, cotton nightdress, and scarlet flannel jacket. It may help "A Lover of Children" and others who read this to realise how gratefully these small garments are received, when they know that all hospital children are provided with two sets of clothing—one for night, and one for day. In this hospital there are 127 in-patients. Therefore, roughly speaking, allowing for the constant entrance and exit of patients, together with the weekly change of linen, 304 nightdresses, vests, and jackets are used weekly.—*K. Philippa Hicks, Lady Superintendent.*

*Nurse E.*—We never prescribe; but plain, wholesome diet has much to do in effecting a cure.

*Ambulance Stations in London.*—Mr. John Harrold will, no doubt, be able to obtain the information he desires on application to Mr. Thomas Ryan, of St. Mary's Hospital, Paddington, W.; or to the hon. sec. of St. John Ambulance Association, Clerkenwell Gate, E.C.

*Inquirer.*—Have your terms printed on the back of your card. When disengaged, leave your card on the doctor's, date it at top, and make it read, Nurse —— begs to inform Dr. —— that she is at present disengaged.

*E. A. A.*—See "Notes and News."

*Constant Reader.*—No big London general hospital will take you at 23 years of age, but Bolton Infirmary will take you for a three years' course of training, or the Brompton Consumption Hospital, or Huddersfield Infirmary, or the North-West London Hospital. If you can afford it, enter for three months first as a paying probationer, for you probably have no notion of the work before you.

*L. B.*—The "Family Physician" is published at 21s. by Cassell and Co., Ludgate Hill, London.

*Trained Midwife.*—The question is one which can only be judged and decided according to the rules of the institution. Surely if the medical officer says "No," the Matron will not insist. Why not bring it before the committee?

*Miss Goodwin.*—A red or blue cross on the shoulder of the uniform cloak would be appropriate, and would infringe on no one's rights.

### Vacancies.

THE following vacancies are announced:

### Matrons.

Luton Cottage Hospital.
Kent County Asylum (assist.); £85.

### Nurses.

Abbey Parochial Hospital, Paisley; £25.

Bath Royal United Hospital; £25.

Bournemouth Institute; £25.

Barony Parochial Hospital, Glasgow (night); £24.

Belfast Royal Hospital (night); £30.

Belfast Home and Training School; £26.

Central London Throat and Ear Hospital; £14.

Croydon Union (night); £22.

Clayton Hospital, Wakefield.

Derby County Asylum (night); £23.

General Institute, Covent Garden.

Hull Home.

Harding Institute, Stoke-on-Trent.

Huddersfield Nurses' Home.

Jessop Hospital, Sheffield; £25.

Leicester Institution; £25.

Luton Cottage Hospital.

Leeds Institution; £14.

Leeds Hospital for Women; £25. Assist., £16; night, £20.

Merthyr Tydfil Workhouse Infirmary; £30.

Manchester Royal Eye Hospital (night).

Middlesboro' Association.

Northern Fever Hospital, N.; £22.

Poplar and Stepney Asylum, Bromley; £21 10s.

P.M.V. Homes, Addlestone, Surrey; £15.

Samaritan Free Hospital, N.W.

Stoke-upon-Trent Workhouse; £20.

St. Pancras Infirmary; £20.

Suffolk General Hospital, Bury St. Edmunds; £20.

Southport Infirmary: £26.

St. Leonard. Shoreditch, £20; temp., £22; assist., £17.

Swansea and South Wales Institute.

Scarboro' Institute and Home Hospital.

Victoria Home, Bournemouth; £25.

Wilson Institution, W.; £30.

Working Infirmary Association.

West Ham Hospital, E.; £24.

Worcester Infirmary (night supt.); £27.

West London Hospital; £24.

### District Nurses.

Bath Nurses' Home; £1 1s. per week.

Jersey Institute; £25.

Middlesboro' Association.

Mansfield District Hospital; £30.

Parish Church, Halifax; £55.

Perth Sick Poor Nursing Society; £25.

### Probationers.

Albert Edward Infirmary, Wigan.

Middlesboro' Association.

Workhouse Infirmary Association

Walsall Cottage Hospital.

St. Helena Home, N.W.

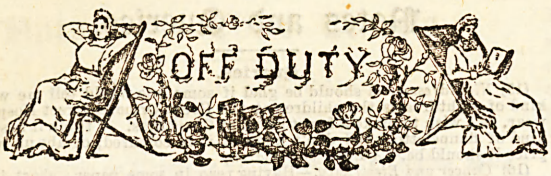

### Our Understandings.

*(Conclusion.)*

The next step was that the ankles and legs needed protection. ". . . . Previous to this the only protection afforded had been the fashion of wrapping the legs with skins or cloths. By the time the shoe had reached this degree of perfection, came the desire of ornamentation," the useful being accomplished, "and scallops of brightly-coloured leather, and finally embroidery became fashionable," When fashion stepped in, of course, vagaries followed. The long peaks of the shoes in the reign of Edward IV. turned upwards from the toes so far that they had to be fastened to the knees by silver chains; then, in a few years, a contrary mode set in; shoes grew so wide that feet resembled huge platters.

Heels, it is said, owe their origin to Persia, where they were introduced upon sandals, in the shape of blocks of wood fixed underneath, such being the root idea of those deformities to which lovely woman owes so many of her woes. A high, unsteady heel, it is an open secret, injures the leg tendons, and affects the spine as well as internal organs, which are liable to be displaced by the thrown-forward position entailed. In Persia, the first home of the heel, however, these blocks of wood were used simply to "raise the feet from the burning sands of that country, and were about two inches high," With the Persian women these blocks were vastly higher than those affected by the men, their height being from eighteen inches to two feet, thus becoming more of the nature of stilts than anything else. Strangely enough, many years after, a similar fashion came into vogue in Venice; but the *motif* in this case was comically different, for "by its means jealous husbands thought they would be able to keep their wives at home." The supports of such shoes in Venice were called "chapineys," and to appease the vanity of the ladies, and doubtless also to sugar the pill, were made highly ornate. The height of these chapineys determined the rank of the wearer, an extra coating for the pill, "the noblest dames being permitted to wear them one half-yard or more high."

We read that in the sixteenth century long-legged boots were worn in France and England, and the boots of the cavaliers were made with commonly wide tops that were rolled over. At first the boot was made with broad legs, for the simple reason that shoemaking had not reached the degree of perfection that turned out a boot with long close fitting legs. From convenience it was but a short step to style, and each "fop of the period was soon trying to outdo his neighbour in the width of his boot-top."

Going from bad to worse in exaggeration, the width of boot-tops in the time of Louis XV. arrived at such a pitch that only bow-legged individuals could wear them with any common degree of comfort! However, Nature does not turn out many such mistakes in her handiwork, so the straight-limbed beings grew tired of "the necessary straddling gait, and began to turn down the top. . . . . From 1550 to 1750, the subject of foot-gear was very prominent, and many extravagances were lavished on unique and costly foot-coverings. Cardinal Wolsey is credited with wearing shoes worth £30,000, while John Spencer wore at his wedding shoes valued at £4,000. Ben Johnson writes of a gallant who

'Wore a farm in shoe-strings, edged with gold,
And spangled garters worth a copyhold.'"

Coming down to the seventeenth century, we find then, and only then, the shoe to be assuming somewhat the shape and mode of the present day. A most practical boot was made, at that time, for postilions, the great feature of which was its excessive heaviness, the foot and ankle being guarded with strips and bands of iron. This was with the object of placing the wearer, should he be unfortunate enough to fall from his horse, in a position to bear the wheels of the carriage passing over his legs without injury.

With the eighteenth century dawned the inspiration, hitherto unthought of, the invention of rights and lefts, for, until the happy thought occurred to an Englishman to construct a separate shoe for each foot, they had been made with the view of suiting either or any; and "from that time forward scientific principles began to be applied to the making of shoes, with due regard to the anatomy of the foot."

Though we of to-day have lost the coloured decorations, the embroideries both golden and silvern, the lace and gay tags of our ancestors, we flatter ourselves upon having gained a symmetry more to be coveted. Given a shapely foot—with an instep—and the boot and shoe of the present moment are, to seeing eyes, "things of beauty, joys for ever."

### Hospital Memories.

Some time ago I had a knock,
And, battered by the nervous shock,
I went straightway to London town;
At Gower Street they put me down,
The hospital received me in,
I had a bathe up to my chin;
One was the number of my ward,
In which I found good bed and board.

But when I saw the glittering knife,
I loudly screamed, "Oh spare my life!
Oh, do not steal my life blood warm,
Or choke my lungs with chloroform!"
Yet soon my fearful eyes grew dim,
And though they gashed my quivering limb,
I now can say with all my heart,
They're welcome to the missing part!

I cried as soon as I grew better,
"Nurse! cut this bandage, 'tis my fetter."
She kindly said, "I'll break your chain,
But mind you don't come here again!"
So, like a prisoner from a cell,
I bounded home quite sound and well.
And I will now conclude this verse
With thanks to doctor and to nurse.—E. C. Newby.

### Amusements and Relaxation.

**N.B.-THIRD QUARTERLY WORD COMPETITION**

**Commenced Oct. 4, 1890; ends Dec. 27, 1890.**

Three prizes of 15s., 10s., 5s., will be given for the largest number of words derived from the words set for dissection.

N.B.—Word dissections must be sent in WEEKLY not later than the first post on Thursday to the *Prize Editor*, 140, Strand, W.C., arranged alphabetically, with correct total affixed.

The word for dissection for this, the NINTH week of the quarter, being "INSTRUMENT."

| Names. | Nov. 20th. | Totals. | Names. | Nov. 20th. | Totals. |
|---|---|---|---|---|---|
| Jenny Wren | 64 | 345 | Checkmate | — | 76 |
| Tinie | — | 55 | Silver King | — | 163 |
| Agamemnon | 63 | 344 | S. Anthony | — | 76 |
| Patience | 63 | 344 | Quackah | — | 75 |
| Ecila | 64 | 345 | Reynard | 62 | 310 |
| Lightowlers | 57 | 324 | Sally | — | 27 |
| Rouge | — | 89 | Success | — | 61 |
| Wyamaris | 65 | 329 | Caledonia | — | 52 |
| Qu'appelle | 60 | 319 | Nurse Emma | 27 | 179 |
| Nosam | 62 | 319 | Hazel | — | 20 |
| Nurse Hilda | — | 44 | Pallas | — | 48 |
| Lady Betty | 59 | 324 | Puss | — | 15 |
| Grenelle | — | 43 | Shakespeare | 49 | 250 |
| Daisy | 46 | 291 | Melita | 56 | 317 |
| H. A. S. | — | 157 | Nora | — | 10 |
| A. B. C. | — | 66 | Elsie | 35 | 35 |
| Liz | 35 | 235 | | | |

